# Intrinsic Enhancement of Dielectric Permittivity in (Nb + In) co-doped TiO_2_ single crystals

**DOI:** 10.1038/s41598-017-05651-z

**Published:** 2017-07-13

**Authors:** Masaru Kawarasaki, Kenji Tanabe, Ichiro Terasaki, Yasuhiro Fujii, Hiroki Taniguchi

**Affiliations:** 1 Department of Physics, Nagoya University, Nagoya, 464-8602 Japan; 2 Department of Physical Sciences, Ritsumeikan University, Kusatsu, 525-8577 Japan

## Abstract

The development of dielectric materials with colossal permittivity is important for the miniaturization of electronic devices and fabrication of high-density energy-storage devices. The electron-pinned defect-dipoles has been recently proposed to boost the permittivity of (Nb + In) co-doped TiO_2_ to 10^5^. However, the follow-up studies suggest an extrinsic contribution to the colossal permittivity from thermally excited carriers. Herein, we demonstrate a marked enhancement in the permittivity of (Nb + In) co-doped TiO_2_ single crystals at sufficiently low temperatures such that the thermally excited carriers are frozen out and exert no influence on the dielectric response. The results indicate that the permittivity attains quadruple of that for pure TiO_2_. This finding suggests that the electron-pinned defect-dipoles add an extra dielectric response to that of the TiO_2_ host matrix. The results offer a novel approach for the development of functional dielectric materials with large permittivity by engineering complex defects into bulk materials.

## Introduction

In addition to resistors and inductors, capacitors based on the dielectric response of materials are among the most fundamental components of electronic devices. Because capacitance is proportional to the dielectric permittivity (ε′) of the material used, increasing the dielectric permittivity directly improves the density of charge accumulation and thereby supports the development of innovative nanoelectronic and power-electronic devices^1, 2^. Several approaches have been proposed for designing large-permittivity materials^3–7^. A standard approach takes advantage of the divergent increase of permittivity in a ferroelectric phase transition^8^. The perovskite-type ferroelectric oxide BaTiO_3_, which has a cubic-to-tetragonal ferroelectric phase transition at 393 K, has been used as a high-density capacitor in applications requiring permittivities of ε′ ~ 10^3^ ^9^. The effect of nanoscale heterogeneity on the phase transitions of ferroelectrics may push the dielectric permittivity as high as 10^4^ over a wide temperature range in ferroelectrics known as relaxors^10–14^. Quantum fluctuation also enhances the dielectric response by competing with ferroelectric ordering in the low-temperature region, as observed in quantum para/ferroelectrics^15–18^. A colossal permittivity close to 10^5^ has been reported for CaCu_3_Ti_4_O_12_, which undergoes no ferroelectric phase transition^19^. The origin of this colossal permittivity was traced to the Maxwell–Wagner effect, which stems from the spatial heterogeneity of conductivity in materials due to various reasons, e.g., charge accumulation around the interfaces between the electrodes and materials and/or the grain boundaries in ceramic materials^20–22^.

The electron-pinned defect-dipole has recently been proposed as a new route to achieve colossal permittivity by engineering complex defects into bulk materials^23^. This effect has been demonstrated in (Nb + In) co-doped TiO_2_, whose dielectric permittivity has been shown to reach a value of 6 × 10^4^ over a frequency range of 10^1^–10^6^ Hz with little dielectric loss. Studies have suggested that a mutually charge-compensating heterovalent substitution of Nb^5+^ and In^3+^ for Ti^4+^ leads to the formation of complex defects in the TiO_2_ host matrix; subsequently, the trapped electrons provide the source of the colossal permittivity. However, the follow-up studies have claimed that the colossal permittivity of (Nb + In) co-doped TiO_2_ has an extrinsic origin in the spatial heterogeneity of conductivity, as in the case of CaCu_3_Ti_4_O_12_^24–26^.

**Figure 1:**
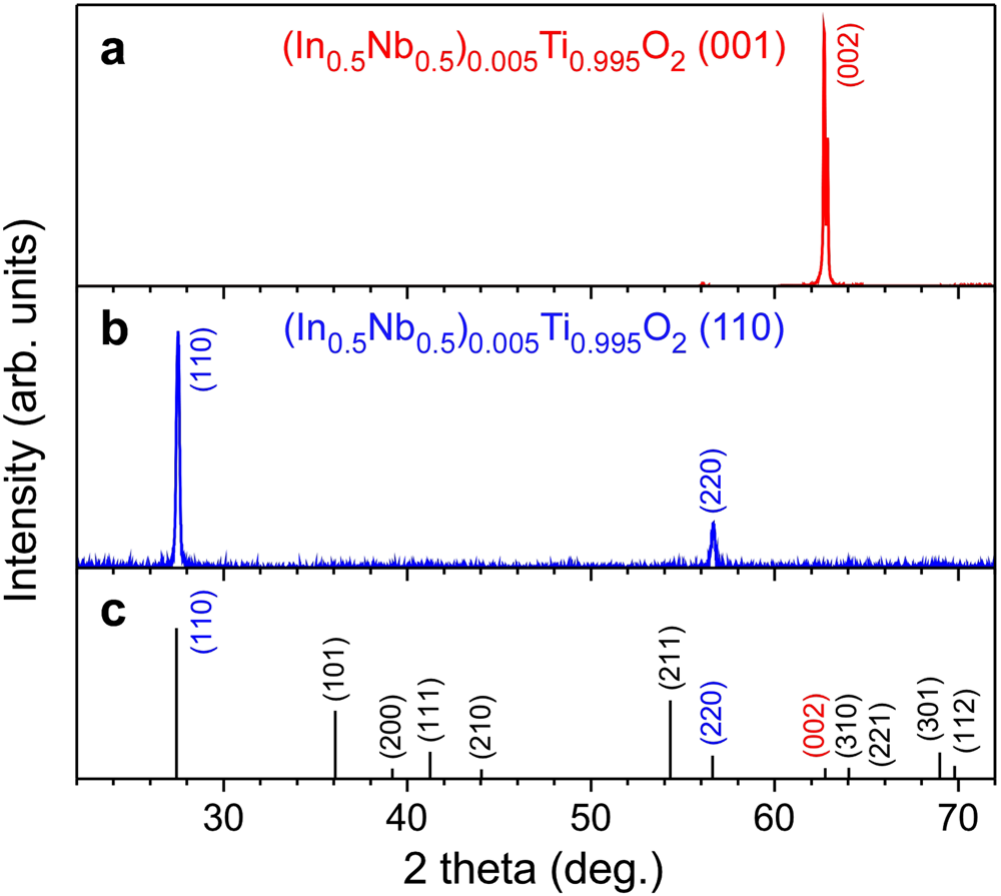
X-ray diffraction patterns of NITO-0.5% single crystals with (**a**) (001) and (**b**) (110) wide surfaces, wherein the incident X-ray was irradiated normal to the wide surface. The calculated diffraction pattern of TiO_2_ is presented in Panel (c).

In this article, we demonstrate an intrinsic enhancement in the dielectric permittivity of (Nb + In) co-doped TiO_2_ using measurements of the single crystals of (Nb_0.5_In_0.5_)_0.005_Ti_0.995_O_2_ (NITO-0.5%), whose orientations were confirmed by x-ray diffraction measurements (Fig. 1). The ε′ ~ 10^5^ permittivity of NITO-0.5% was found to decay in the low-temperature region, suggesting that thermally excited electrons accumulate at the sample-electrode interfaces in the high-temperature region and affect the Maxwell–Wagner-type colossal permittivity. We found that at a temperature of approximately 2 K, the permittivity of NITO-0.5% was significantly larger than that of pure TiO_2_; however, at this temperature, the contribution of the thermally excited electrons is expected to be suppressed completely. The permittivity of NITO-0.5% at this temperature also showed strong anisotropy, with values of ε′ reaching 900 and 250 along the [001] and [110] directions, respectively. The corresponding values for the pure TiO_2_ were 260 and 120 at this low temperature. Our findings suggest that complex-defect engineering can be used to increase the dielectric permittivity of materials.

**Figure 2:**
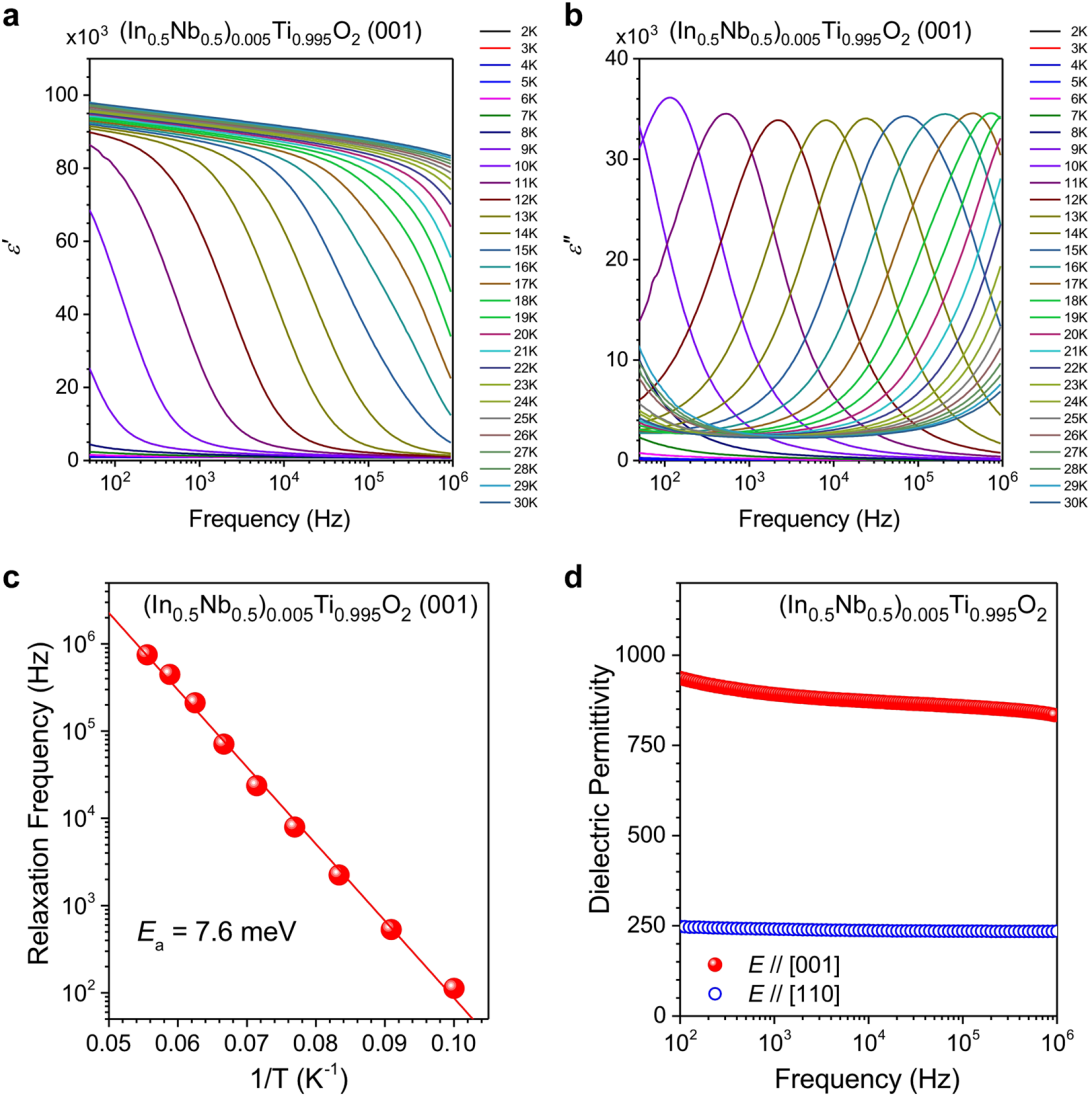
Frequency dispersions for (**a**) the real and (**b**) imaginary parts of the dielectric permittivity of a NITO-0.5% single crystal with a (001) wide surface measured at several temperatures ranging from 2 K to 30 K. Panel (c) shows an Arrhenius plot for the relaxation frequency against the inverse temperature. The activation energy of 7.6 meV for the thermally excited carriers was estimated from linear fitting and is shown by the solid line. Panel (d) shows the frequency dispersions for the dielectric permittivity in NITO-0.5% at 4 K measured along the [001] and the [110] directions, which are represented by closed and open circles, respectively.

Figure 2a shows the real part of the complex dielectric permittivity ε′ of NITO-0.5% measured along a direction normal to the (001) plane at several temperatures and plotted as a function of the measurement frequency. At 30 K, the highest temperature used in these measurements, the value of ε′ was close to 10^5^ over the entire frequency range of 10^2^–10^6^ Hz and only little frequency dispersion was observed. Since the measurements were performed on a single crystal, it is plausible that the colossal permittivity originated from the Maxwell–Wagner effect of charge accumulation around the interfaces between the sample and electrodes^26^. According to the surface barrier layer capacitor (SBLC) model, the real part of dielectric permittivity ε′ in the low-frequency region decreases with increasing a testing voltage for dielectric measurements, whereas the imaginary part increases conversely^26–28^. An observed testing-voltage-dependence in the NITO-0.5% agreed qualitatively with the SBLC model (Supplemental Fig. 1), thereby supporting an extrinsic influence from the interfaces on the dielectric response of NITO-0.5%. It should be noted here that we observed a non-monotonic testing-voltage-dependence in the real part of dielectric permittivity as presented in the inset of the top panel of Supplemental Fig. 1. This behavior is discussed later in this paragraph. The colossal permittivity of NITO-0.5% due to the extrinsic Maxwell–Wagner effect rapidly disappeared as the temperature decreased, accompanied by a downward shift in the dielectric relaxation frequency, as shown in Fig. 2a. A slowing of the dielectric response with cooling can be clearly seen in the temperature dependence of the characteristic peak shown in Fig. 2b, which represents the frequency dispersion of the imaginary part of the complex permittivity ε″. The activation energy *E*_a_ of the thermally excited carriers, which contributes to the colossal permittivity, was estimated to be 7.6 meV using the Arrhenius plot shown in Fig. 2c. This figure shows the logarithmic plot of the peak frequencies of ε″ against the inverse of the temperature. Note that, since the functional form of relaxation was not known, we estimated the peak frequencies from peak values of the relaxation profiles. In the high temperature region where the colossal permittivity was observed, we found an additional increase in the real and imaginary parts of permittivity in the low frequency region (Supplemental Fig. 2). This component would be caused by a leakage current in the sample. An extrinsic influence of the leakage current on the dielectric response is characterized by a Drude-type frequency dispersion, which has a maximum at zero-frequency for both the real and the imaginary parts of dielectric permittivity. Since the leakage current increases as the testing voltage increases, both the real and the imaginary parts of the permittivity increase on elevating the testing voltage. This testing-voltage-dependence is different from that for SBLC; on increasing the testing voltage, the real part of dielectric permittivity increases for the leakage current, whereas it decreases for SBLC. The non-trivial testing-voltage-dependence in the real part of the permittivity observed in the present study would be due to superposition of these mutually opposite effects, thus suggesting a multiple contribution of dielectric response in the high temperature region: the effects of SBLC and the leakage current, both of which are induced by the thermally excited carriers. Note here that tanδ ( = ε″/ε′) of NITO-0.5, which was measured at 1 MHz in the present study, reaches 0.37 at room temperature. This value is an order of magnitude greater than that reported in ref. 23. This difference implies diversity of polarization mechanism in the (In + Nb) co-doped TiO_2_. The polarization mechanism in the high temperature region is possibly very sensitive for synthesis conditions.

As can be seen in the low-temperature region of Fig. 2a, when the extrinsic colossal permittivity disappears completely, the single crystal of NITO-0.5% still has a permittivity close to 10^3^ along the [001] direction with no dielectric relaxation in the frequency range of 10^2^–10^6^ Hz. This value is much larger than that of pure TiO_2_, whose permittivity is ~270 along the [001] direction at low temperatures^29^. As conductive electrons are very weakly excited at 2 K, the marked enhancement of permittivity observed here must have stemmed from the additional electronic states induced by the co-doping of Nb^5+^ and In^3+^. This supports the concept of using electron-pinned defect-dipoles to achieve a large permittivity. Furthermore, the dielectric permittivity of NITO-0.5% in the low-temperature region was found to be strongly anisotropic. The dielectric dispersions measured at 4 K for samples with (001) and (110) wide surfaces are shown by the closed and open circles, respectively, in Fig. 2d. As can be seen, the dielectric permittivity along the [110] direction is 250 over the full frequency range observed herein whereas that along the [001] direction is 900. Moreover, the dielectric permittivity along the [110] axis is much larger than that of pure TiO_2_ (ε′//[110] ∼ 120)^29^. Note that the dielectric permittivity of NITO-10% single crystal along [110] direction was previously reported to be ~100^26^. This discrepancy would be caused by differences in a concentration of dopants and growth conditions for the present study and the previous report.

**Figure 3:**
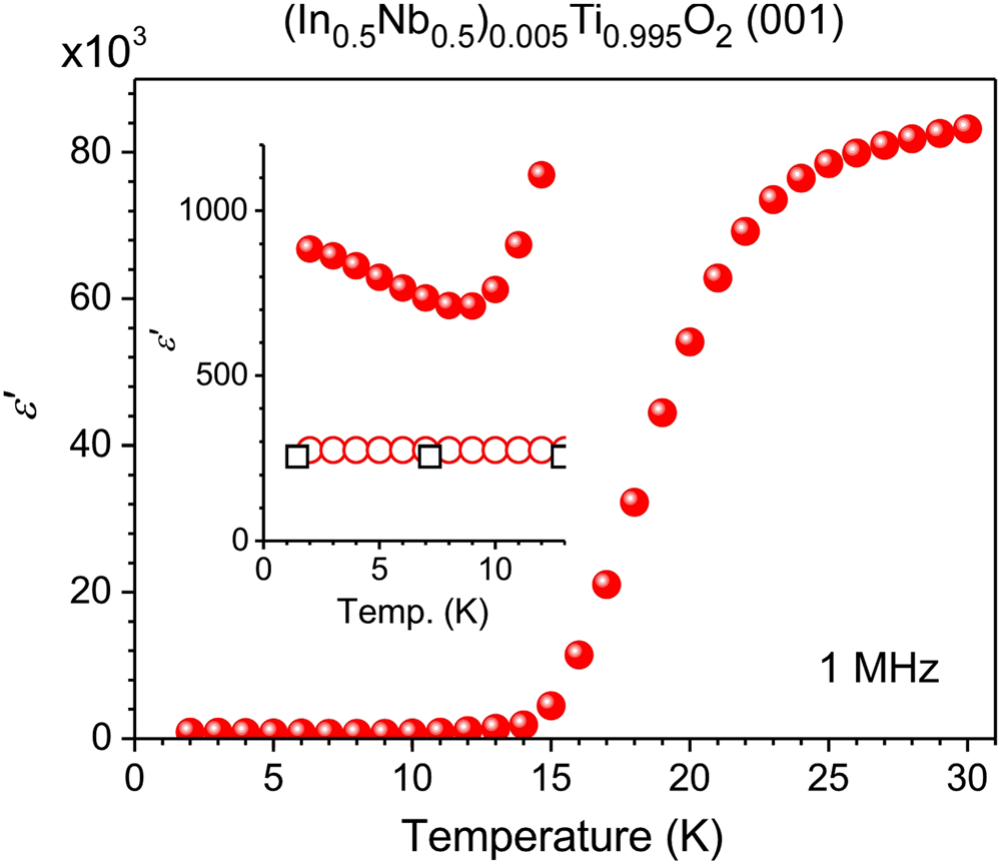
Real part of the dielectric permittivity of a NITO-0.5% single crystal with a (001) wide surface measured at 1 MHz and shown as a function of temperature by closed circles. The inset shows a magnified view of the temperature region below 13 K. The open circles indicate the real part of the dielectric permittivity of pure TiO_2_ observed in the present study. The previous result of the real part of the dielectric permittivity of pure TiO_2_ in ref. 29 are plotted by the open squares for comparison.

The variation in the dielectric permittivity of NITO-0.5% along the [001] direction at 1 MHz is shown as a function of temperature by the solid circles in Fig. 3. The inset shows a magnified view of the temperature region below 13 K. The temperature dependence of the dielectric permittivity of pure TiO_2_ along the [001] direction is also plotted in the inset, where open circles denote the results of the present study and squares denote those reported in ref. 29. The extrinsic colossal permittivity rapidly decays as the temperature decreases before finally disappearing completely at ~13 K. As shown in the inset, under further cooling, the permittivity begins to increase as the temperature decreases. This intrinsic behavior of NITO-0.5% cannot be caused by a Maxwell–Wagner-type dielectric response because the thermally excited electrons freeze at these temperatures. Pure TiO_2_ is known to show similar behavior across the extended temperature region due to a so-called incipient ferroelectricity. The increase permittivity observed in NITO-0.5 near 0 K would be a trace of the incipient ferroelectricity as in the case of the pure TiO_2_. We next compare the temperature dependence of the intrinsic dielectric permittivity of NITO-0.5% with that of pure TiO_2_.

**Figure 4:**
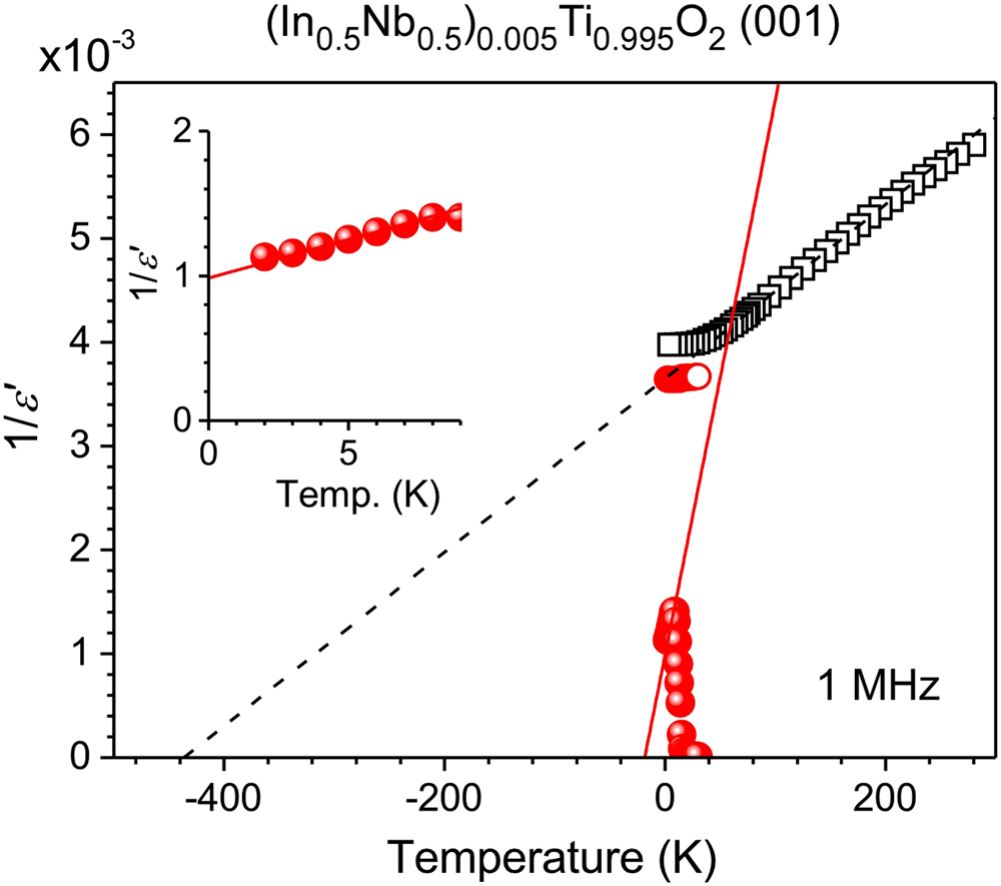
Inverse permittivity of a NITO-0.5% single crystal with a (001) wide surface measured at 1 MHz and plotted as a function of temperature by closed circles. Open circles and squares denote the results for pure TiO_2_ obtained in the present study and those reported in ref. 30. The solid and broken lines indicate the extrapolated temperature dependences of NITO-0.5% and pure TiO_2_. These were obtained by fitting the linear parts of the plots using the Curie–Weiss law to estimate the virtual phase transition temperatures. The inset shows a magnified view of the temperature region below 9 K.

Figure 4 shows the temperature dependence of the inverse permittivity of NITO-0.5% (closed circles) and pure TiO_2_ (open circles). The inset shows a magnified view of the temperature region from 0 K to 9 K. Open circles and squares are used to compare the results of the present study with those reported in ref. 30. The plot with the open squares shows a monotonic decrease in the inverse permittivity of pure TiO_2_ as the temperature decreases. This is caused by the incipient ferroelectricity in pure TiO_2_, with the system approaching a ferroelectric phase transition as it cools; however, this does not occur in a finite temperature. The virtual phase transition temperature of pure TiO_2_ can be estimated by extrapolating the temperature dependence of the inverse permittivity using the Curie–Weiss law. Note that a virtual phase transition temperature has been often used to characterize strength of ferroelectric interactions in a quantum paraelectrics and an incipient ferroelectrics^15^. It sometimes takes a negative value when the ferroelectric interaction is weak; For instance, the incipient ferroelectric CaTiO_3_ has the negative transition temperature of −111 K^31^. The broken line in the figure shows the extrapolation, suggesting a virtual transition temperature of −435 K for pure TiO_2_. One possible explanation for the permittivity enhancement in NITO-0.5% is that co-doping of Nb^5+^ and In^3+^ shifts the virtual transition temperature of TiO_2_ to a higher temperature and thereby raises the dielectric permittivity. This is often observed in quantum paraelectric and quantum paraelectric-like materials^15–18, 31, 32^. To investigate this hypothesis, the dielectric permittivity of NITO-0.5% below 13 K was also extrapolated using the Curie–Weiss law. The results are shown in Fig. 4 and its inset. The extrapolation estimated a virtual transition temperature of −19 K, an increase of no less than 400 K. Such a large shift seems implausible for the amount of dopant present in NITO-0.5%. Note that, as presented in Supplemental Fig. 3, no change in lattice dynamics of TiO_2_ due to the (Nb + In) co-doping was detected in the present Raman scattering experiments at low temperatures, ruling out a possibility of the enhanced ionic polarizability for the origin of enhanced permittivity in NITO-0.5% in the low temperature region.

Thus, the most plausible origin of the large permittivity of NITO-0.5% is response of electrons at the complex defects. Assuming that the difference in the permittivity between the NITO-0.5% and pure TiO_2_ at 4 K, i.e., Δε′ = 640, stems from the response of electrons at the complex defects, an induced polarization of 5.66 × 10^−5^ Cm^−2^ will be required at the present testing voltage of 100 V/cm. The defect density of NITO-0.5%, assuming one complex defect every 200 unit cells, is estimated to be ~1.6 × 10^20^ cm^−3^. An averaged dipole moment of 3.54 × 10^−31^ Cm for a single complex defect is then estimated from the value of the induced polarization. The induced dipole moment estimated here corresponds to a reduced displacement of the elementary charge of ~0.02 Å under a voltage of 100 V/cm. This estimated reduced displacement is equivalent to several percents of the size of a complex defect, which is considered to spread over few Å^23^. The complex defects proposed in ref. 23 have forms of ${{\rm{In}}}_{2}^{3+}{{\rm{V}}}_{\ddot{{\rm{O}}}}{{\rm{Ti}}}^{3+}$ triangle and ${{\rm{Nb}}}_{2}^{5+}{{\rm{Ti}}}^{3+}{{\rm{A}}}_{{\rm{Ti}}}$ diamond, whose configurations are closely associated with an anisotropic crystal structure of TiO_2_. The strong anisotropy in the dielectric response at the low temperature, which has been found in the present study, would thus be caused by the anisotropy in the structures of complex defects in NITO. To increase the complex-defect-induced permittivity further, the defect density must be increased and/or the defect state should be made dispersive. However, such modifications would require a tradeoff with the insulating properties of the material. A next step would be to optimize the defect-induced dielectric response via the design of the electronic states in the complex defects.

Although further quantitative investigation is required, the results of the present study demonstrate that co-doping of Nb^5+^ and In^3+^ introduces an additional agent that responds to the external electric field in the host TiO_2_ matrix and thereby enhances its intrinsic dielectric permittivity. We believe that this work elucidates the potential of defect engineering in increasing the dielectric permittivity of a host matrix and contributing to the development of innovative nanoelectronic and power-electronic devices.

## Methods

Stoichiometric mixtures of TiO_2_(4 N), In_2_O_3_(3 N), and Nb_2_O_5_(4 N) were subjected to hydrostatic pressing to produce rods in crystal growth. These rods were then sintered at 1673 K for 10 h in a box furnace. Single crystals were grown using an optical floating zone furnace (FZ-T-4000-H-I-V; Crystal Systems) equipped with a single ellipsoidal gold-plated mirror. The obtained single crystals of NITO-0.5% were sliced using a diamond cutter to prepare two samples with (001) and (110) wide surfaces. The (001) and (110) orientations of the obtained samples were confirmed using X-ray diffraction (RIGAKU RINT-2000), as shown in Fig. 1. Dielectric permittivity was measured using a Quantum Design Physical Property Measurement System equipped with a Keysight 4284 A precision LCR-meter in the temperature region from 2 K to 30 K.

## Electronic supplementary material

Intrinsic Enhancement of Dielectric Permittivity in (Nb + In) co-doped TiO2 single crystals
