# Supplementary material for: Intrinsic Enhancement of Dielectric Permittivity in (Nb + In) co-doped TiO2 single crystals
Source: Sci Rep. 2017 Jul 13;7:5351. doi: 10.1038/s41598-017-05651-z (PMC5509748; doi:10.1038/s41598-017-05651-z)
Supplement: Supplementary file 1 — Intrinsic Enhancement of Dielectric Permittivity in (Nb + In) co-doped TiO2 single crystals [file 41598_2017_5651_MOESM1_ESM.pdf]

Intrinsic Enhancement of Dielectric Permittivity in (Nb + In) co-doped TiO_2 single crystals

Masaru Kawarasaki[1], Kenji Tanabe[1], Ichiro Terasaki[1], Yasuhiro Fujii[2], and Hiroki Taniguchi[1*]

[1]Department of Physics, Nagoya University, Nagoya 464-8602, Japan.
[2]Department of Physical Sciences, Ritsumeikan University, Kusatsu 525-8577, Japan
[*]e-mail: hiroki_taniguchi@cc.nagoya-u.ac.jp

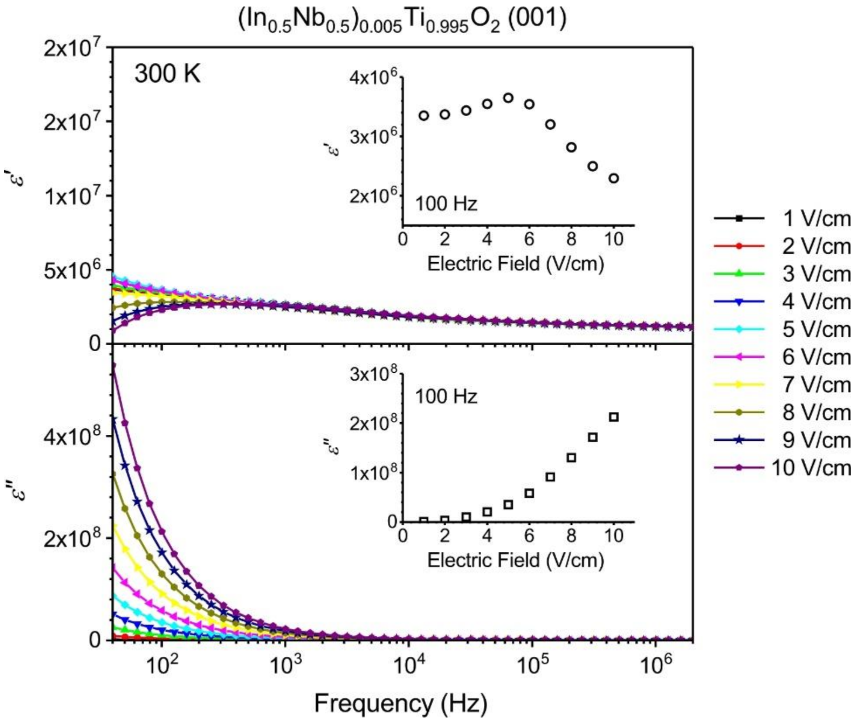

Supplemental Figure 1: Testing-voltage-dependences for (top) the real and (bottom) the imaginary parts of dielectric permittivity of a NITO-0.5% single crystal with a (001) wide surface measured at 300 K. Insets in the top and bottom panels present real and imaginary parts of dielectric permittivity at 100 Hz as a function of the applied electric field plotted by open circles and open squares, respectively.

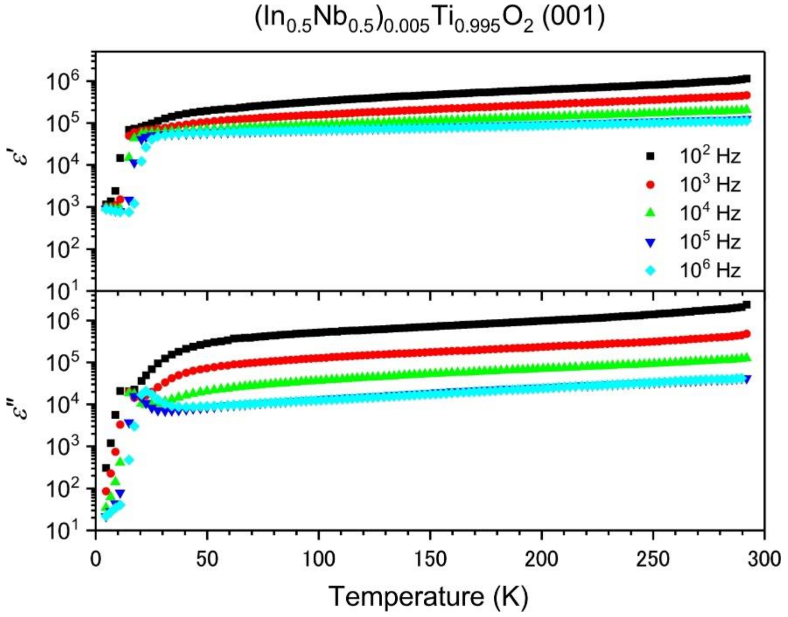

Supplemental Figure 2: Temperature dependences for (top) the real and (bottom) the imaginary parts of dielectric permittivity of a NITO-0.5% single crystal with a (001) wide surface measured at several frequencies.

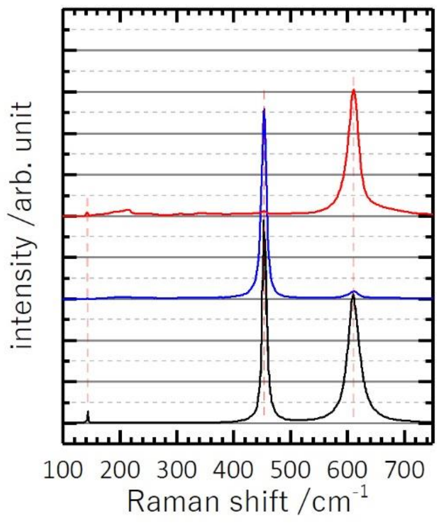

Supplemental Figure 3: Comparison of the Raman spectra of NITO-0.5 and pure rutile. The upper red curve and middle blue one show polarized(VV) and depolarized (VH) spectra of NITO-0.5 (110)-platelet at 3 K, respectively. The lower black curve represents the literature data of pure rutile obtained from Fig. 1 of [34]. The dashed lines show the Raman shift of each peak. Raman measurements were performed with a backscattering geometry. The temperature of the specimen was controlled using a Gifford-McMahon cryostat (Thermal-Block SB-4KCRS). A frequency doubled diode-pumped solid-state laser operating at 532 nm (Oxxius LMX-300S) was employed as the excitation source. The excitation light with a power of ~5mW was focused to the sample using a 5x objective lens, and the scattered light was corrected by the same lens. The Raman scattered light was analyzed by a single monochromator (Jobin-Yvon HR320, 1200gr/mm) equipped with a CCD camera(Andor DU420), while the elastic stray light was rejected using three ultra-narrow band holographic notch filters (OptiGrate). [35]

[34] Betsch, R. J., Park, H. L. & White, W. B. Raman spectra of stoichiometric and defect rutile. *Mat. Res. Bull.* **26**, 613 (1991).

[35] Fujii, Y., Katayama, D. & Koreeda, A. Broadband light scattering spectroscopy utilizing an ultra-narrowband holographic notch filter. *Jpn. J. Appl. Phys.* **55**, 10TC03 (2016).
